# A computational model to investigate assumptions in the headturn preference procedure

**DOI:** 10.3389/fpsyg.2013.00676

**Published:** 2013-10-04

**Authors:** Christina Bergmann, Louis ten Bosch, Paula Fikkert, Lou Boves

**Affiliations:** ^1^Centre for Language Studies, Radboud University NijmegenNijmegen, Netherlands; ^2^International Max Planck Research School for Language Sciences, Max Planck Institute for Psycholinguistics, Radboud University NijmegenNijmegen, Netherlands; ^3^Centre for Language and Speech Technology, Radboud University NijmegenNijmegen, Netherlands

**Keywords:** headturn preference procedure, language acquisition, segmentation, attention, speech processing

## Abstract

In this paper we use a computational model to investigate four assumptions that are tacitly present in interpreting the results of studies on infants' speech processing abilities using the Headturn Preference Procedure (HPP): (1) behavioral differences originate in different processing; (2) processing involves some form of recognition; (3) words are segmented from connected speech; and (4) differences between infants should not affect overall results. In addition, we investigate the impact of two potentially important aspects in the design and execution of the experiments: (a) the specific voices used in the two parts on HPP experiments (familiarization and test) and (b) the experimenter's criterion for what is a sufficient headturn angle. The model is designed to be maximize cognitive plausibility. It takes real speech as input, and it contains a module that converts the output of internal speech processing and recognition into headturns that can yield real-time listening preference measurements. Internal processing is based on distributed episodic representations in combination with a matching procedure based on the assumptions that complex episodes can be decomposed as positive weighted sums of simpler constituents. Model simulations show that the first assumptions hold under two different definitions of recognition. However, explicit segmentation is not necessary to simulate the behaviors observed in infant studies. Differences in attention span between infants can affect the outcomes of an experiment. The same holds for the experimenter's decision criterion. The speakers used in experiments affect outcomes in complex ways that require further investigation. The paper ends with recommendations for future studies using the HPP.

## 1. Introduction

Infants begin to acquire what will become their native language long before they produce meaningful speech themselves. The last decades have seen a substantial growth in experimental studies that explore this pre-verbal phase of language acquisition, with a particular focus on how infants process speech input. The advent of behavioral research paradigms that tap into infants' underlying cognitive abilities made this research line possible. The paradigms recruit actions infants can readily perform in their daily lives. The prime example of such a paradigm is the Headturn Preference Procedure (HPP), which uses the eponymous headturns to investigate speech processing.

The HPP is based on the observation that infants tend to turn their heads toward interesting events. The time this headturn in maintained is interpreted as infants' amount of interest. Jusczyk and Aslin (1995) demonstrated how the HPP can be used to investigate infants' ability to memorize and recognize speech^1^. A common version of the HPP, as used by Jusczyk and Aslin, typically has two phases. In an initial familiarization phase, infants are exposed to words spoken in isolation. In the test phase that immediately follows familiarization, infants listen to sentences that contain either one of the previously heard words or an unfamiliar word. Differences in the time the head is turned toward each of the two types of test stimuli indicate that infants process test stimuli with and without familiar words differently. Jusczyk and Aslin interpreted such listening time differences as the ability of the infants to discover that the familiarized words are present in some of the test sentences.

Following the seminal work by Jusczyk and Aslin (1995), numerous studies have utilized the HPP to investigate infants' emerging speech processing abilities. Almost invariably, HPP studies use the familiarization-followed-by-test design briefly outlined above, where listening time during the test phase is the behavioral measure (c.f., Section 2 for further details). Subsequent studies have replicated the original finding with infants learning French (Nazzi et al., 2013), Spanish (Bosch et al., 2013), and many other languages. Others have used the HPP to shed light on the influence of various extra-linguistic factors in the processing of speech signals. A number of studies showed that infants cannot readily detect the familiarized words in the test sentences if there are large acoustic differences between familiarization and test phase, for example, when they differ in mood, accent, and gender of the speaker (Houston and Jusczyk, 2000, 2003; Singh et al., 2004; Schmale and Seidl, 2009; Schmale et al., 2010)^2^.

Although there are few published reports of null-results, failures to replicate the outcome of published HPP experiments are not uncommon (see Ferguson and Heene, 2012; for the bias against publishing papers that report failures to replicate). Furthermore, seemingly comparable studies can yield results that support contradicting interpretations. For example, Houston and Jusczyk (2000) tested infants' ability to detect words spoken by one speaker during familiarization in test passages that were spoken by a different speaker. Thereby the authors investigated is whether infants are able to generalize across speakers. The results showed that infants only listened longer to test stimuli containing familiarized words than to test stimuli with novel words if the speakers' gender matched between familiarization and test phase. In a seemingly comparable study, van Heugten and Johnson (2012) found that gender differences do not seem to matter for infants of the same age as tested by Houston and Jusczyk. In addition, the infants in the study by van Heugten and Johnson showed a novelty preference, where infants listened longer to test stimuli without the familiarized words, while Houston and Jusczyk found a familiarity preference.

It is not yet entirely clear which factors exactly determine the behavior of infants in HPP studies (Houston-Price and Nakai, 2004; Aslin, 2007; van Heugten and Johnson, 2012; Nazzi et al., 2013). Studies using the HPP vary in several aspects, including the stimulus material and implementation details. For example, different speakers are used to record stimuli across experiments, and potentially relevant properties of the stimuli (such as voice characteristics) are difficult to report in a meaningful way. Sharing stimulus material among research groups would be an improvement, but is often not feasible unless infants are acquiring the same language (c.f., Nazzi et al.). Differences in implementation are exemplified by seemingly varying criteria for a sufficient headturn, ranging from “at least 30° in the direction of the loudspeaker” (Jusczyk and Aslin, 1995, p. 8) to “at least 70° toward the flashing light” (Hollich, 2006, p. 7). It is possible that such differences in assessment criteria, even if used systematically and accurately, can cause conflicting results.

In addition to these practical issues with HPP studies, there is a more fundamental question that urgently needs attention. In behavioral paradigms, including the HPP, the cognitive processes of interest must be inferred from observable behavior, and these inferences rely on numerous assumptions about the link between overt behavior and cognitive processes. Most behavioral data are compatible with different, perhaps conflicting, assumptions and interpretations (Frank and Tenenbaum, 2011). The present paper addresses these practical and fundamental issues by using a computational model that simulates the test situation of the HPP. The use of a computational model allows for the investigation of fundamental issues, because the implementation of the procedure makes crucial assumptions explicit, and model simulations make it possible to assess whether these assumptions are necessary to simulate infant behavior. At the same time simulations allow us to study the impact of differences in stimulus material and in the practical implementation of the HPP. Although the model is — by necessity — a simplified analogue of an infant (or a group of infants) in an HPP experiment, we aim for its operations and representations to be as cognitively plausible as possible. In consequence, the model simulations can help to better understand the outcome of HPP experiments.

The remainder of this paper is organized as follows: In Section 2 we first describe the HPP in detail along with the assumptions that are commonly made in the interpretation of the results in infant studies before we introduce our computational model in Section 3. We explain how the model makes it possible to test the assumptions discussed in Section 2.1. In addition, we outline how the model is built to maximize cognitive plausibility. The design of the experiments that allow us to investigate the impact of the stimulus material and details of how HPP experiments are conducted is further elaborated on in Section 4. Section 5 presents the results of our experiments. The paper concludes with a general discussion and outlines the implications of the modeling results for the interpretation of results reported in infant studies.

## 2. The headturn preference procedure

**Figure 1:**
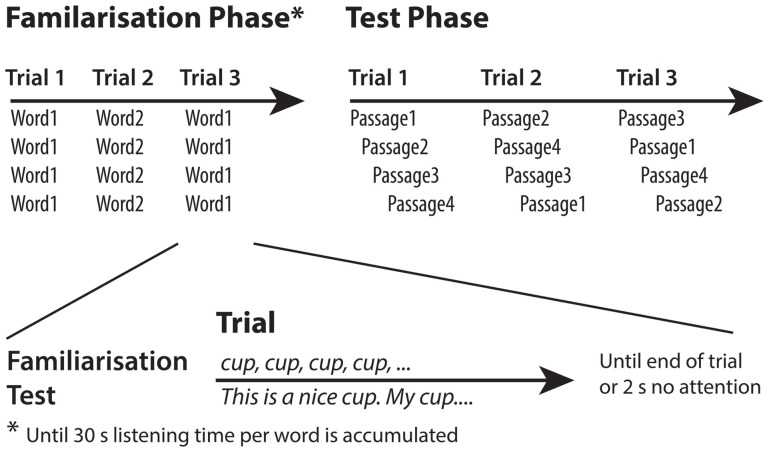
**Exemplary outline of a two-phase headturn experiment**. Infants first hear words spoken in isolation and then listen to sentences that do or do not contain these words.

HPP experiments typically consist of two consecutive phases, as Figure 1 illustrates using an example from the experiments by Jusczyk and Aslin (1995). In the first phase an infant is familiarized with a specific audio(-visual) phenomenon (here: spoken words and the accompanying flashing lamp). The criterion for familiarization is usually a cumulative listening time of at least 30 s for each word. When the familiarization criterion is met the second phase immediately commences. In this phase the infant's reaction to test stimuli is measured that either contain the two familiarized words or two novel words^3^.

In the study of Jusczyk and Aslin (1995), infants were familiarized with two words spoken in isolation (either *cup* and *dog*, or *feet* and *bike*). In the test phase passages of six sentences containing one of the four words were presented in each trial^4^. The infants listened longer to passages containing words with which they were familiarized, as indicated by their maintained headturns (see below for details). Hence, infants showed sufficient memory and processing abilities to store and detect words and to overcome an acoustic difference between embedded and isolated words. Based on their results Jusczyk and Aslin concluded that infants have segmented the passages into smaller chunks and detected the embedded words.

**Figure 2:**
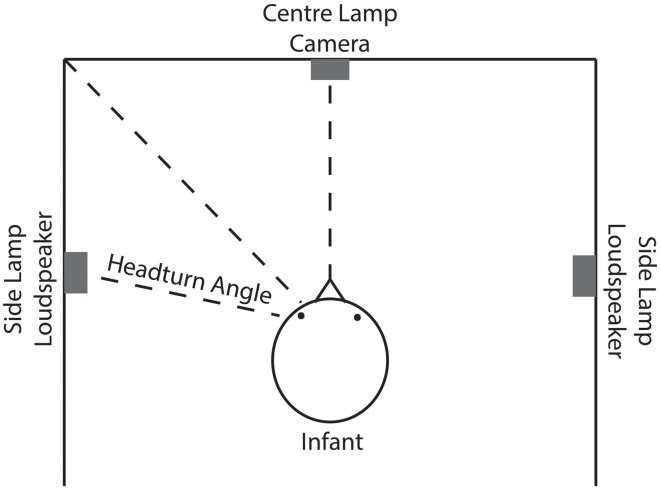
**Schematic outline of the experimental set-up in headturn studies**. The infant is placed in a three-sided booth with lamps on each side and loudspeakers to the left and right. Through a frontal camera, the headturns are observed by the experimenter.

The rationale behind the HPP is that the time an infant spends with the head turned toward a side lamp while presumably listening to speech stimuli coming from that same side indicates the infant's interest in the stimuli. The experimental set-up based on this rationale is depicted in Figure 2. Infants are placed in a three-sided booth with lamps on each wall, one in front of the infant and one on each side. A loudspeaker is mounted beneath each side lamp. Through a video camera facing the infant, the experimenter observes the infant's movements and controls the experiment. A trial starts with the center lamp flashing. As soon as the infant attends to that lamp by turning toward it, one of the side lamps begins to flash, and the central lamp turns off. When the infant turns her head to the side lamp by a pre-determined angle off-center, speech stimuli begin to play from the loudspeaker beneath the flashing side lamp. As long as the head is turned toward the side lamp, the trial continues. Turning the head away for more than 2 consecutive seconds ends the trial prematurely. If the infant turns her head back toward the lamp before 2 s have elapsed the trial is not ended. The time during which the head was turned away is not measured as listening time. Importantly, while headturn angle is a continuous variable, it is converted into a binary criterion by the experimenter: the head is, or is not, turned sufficiently toward the side lamp and the loudspeaker at any moment throughout the trial. The side of the flashing lamp and of presenting the speech stimuli is counterbalanced and bears no relation to the type of trial.

### 2.1. Assumptions in the headturn preference procedure

The HPP aims to tap into infants' linguistic abilities by inferring cognitive processes (in particular speech processing) from observable behavior. Linking overt behavior in HPP experiments to infants' underlying cognitive processes is based on at least four main (implicit) assumptions, which are not straightforward to test experimentally.

First, a listening preference for one type of test stimulus stems from some form of underlying *recognition* of recently heard words. In their seminal work, Jusczyk and Aslin (1995) equate recognition with the detection of a sufficiently high degree of similarity between perceived sound patterns. In a two-phase HPP experiment, presumably unknown words are presented to the infant during familiarization, and then two sets of previously unknown words are compared in testing (one familiarized and one novel). It is thus measured how infants react to words that were recently presented in comparison to entirely novel words.

Second, systematic differences in listening time to passages containing familiar or novel words are due to systematic internal processing differences. Infants' behavior in HPP studies is assumed to be resulting from several processing steps: infants have to internally process speech input and match it to representations stored in internal memory. The memory contains representations of experience before the lab visit as well as representations stored during the familiarization phase, whereas the focus lies on the memorization of familiarized items.

Third, recognition of words in passages, while those words were presented in isolation during familiarization, requires infants to be able to segment words from continuous speech prior to matching. Segmentation entails the chunking of speech into smaller parts and representing those constituents independently.

Fourth, differences between individual infants do not affect the outcome of an experiment, as the main comparison (listening to novel or familiar test stimuli) takes place within participants. This assumption mainly concerns infant-specific factors independent of their linguistic abilities.

## 3. Modeling the headturn preference procedure

First we outline how the model architecture and the simulations aim to address the assumptions discussed in Section 2.1. The model subscribes to the first two assumptions. Following the first assumption, recognition is implemented in the model in the form of a matching process which compares test items to the familiarized stimuli along with a form of past experience. The contents of the memory that the matching process works on are described in Section 3.3, the matching process that operates on the memory is explained in detail in Section 3.4. Section 3.5 lays out how recognition can be implemented. In accordance with the second assumption, the matching procedure should yield systematically different outcomes that signify the model's internal ability to distinguish novel and familiar test items. Based on the outcome of the matching procedure, headturns are simulated. The conversion of internal recognition into overt behavior is discussed in Section 3.6. The third assumption will be assessed by our model. The claim that infants are able to segment words from continuous speech utterances seems unnecessarily strong. A strong segmentation procedure is difficult to implement without assuming that the model decodes and memorizes speech in the form of sequences of discrete linguistic units (such as syllables and phonemes), an ability that infants are still in the process of acquiring (Kuhl, 2004; Newman, 2008). Therefore, we follow the proposal that infants are able to divide a passage consisting of a sequence of six naturally spoken utterances, separated by clear pauses, into the constituting sentences (Hirsh-Pasek et al., 1987; Jusczyk, 1998). The model thus receives its test input in the form of complete sentences, as Sections 3.2 and 3.3 describe. If the model is able to distinguish familiar from novel test items, we show that segmentation is not necessary in the two-phase HPP studies simulated in the present paper. We will investigate the fourth assumption that differences between individual infants do not affect the outcome of an experiment. The role of an infant-dependent parameter that transforms internal recognition into overt headturns will be investigated to this end (see Section 3.6 for further detail).

Simulations with varying criteria for a sufficient degree of headturn assess the impact of implementation details. Furthermore, we use speech produced by four speakers to address the role of the stimulus material in HPP experiments and the model's ability to generalize across speakers. These issues will be explained in more detail in Sections 3.7 and 4.

### 3.1. The model architecture

**Figure 3:**
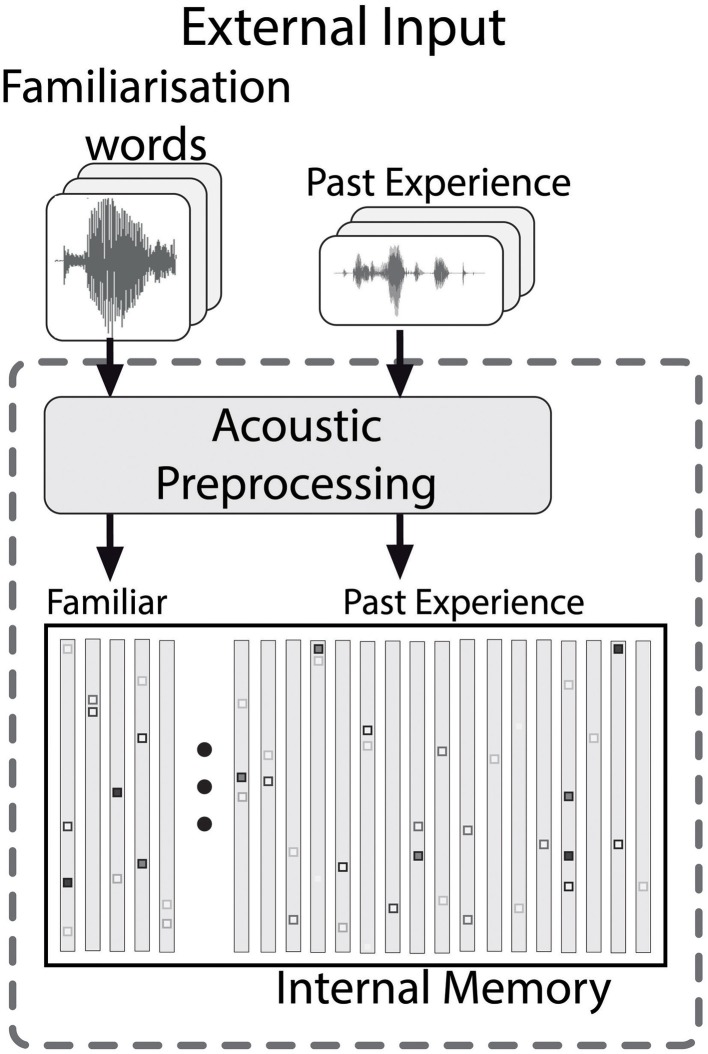
**The memory structure of the model, which contains both the familiarized items and past experience**. Acoustic preprocessing is applied to all contents of the memory.

We developed a computational model that, despite the necessary simplifications, is as cognitively plausible as possible. The model contains general purpose processing skills which infants would also need for other tasks. The architecture of the model during the familiarization phase is shown in Figure 3. All input consists of real speech that proceeds through a sequence of processing steps, which are explained in detail in the following sections. In the model, the familiarization phase is simulated by storing the stimuli in an internal model memory that is already populated by episodic representations of speech (and sounds) that the modeled infant heard before the lab visit (Goldinger, 1998). The details of the model memory are described in Section 3.3.

**Figure 4:**
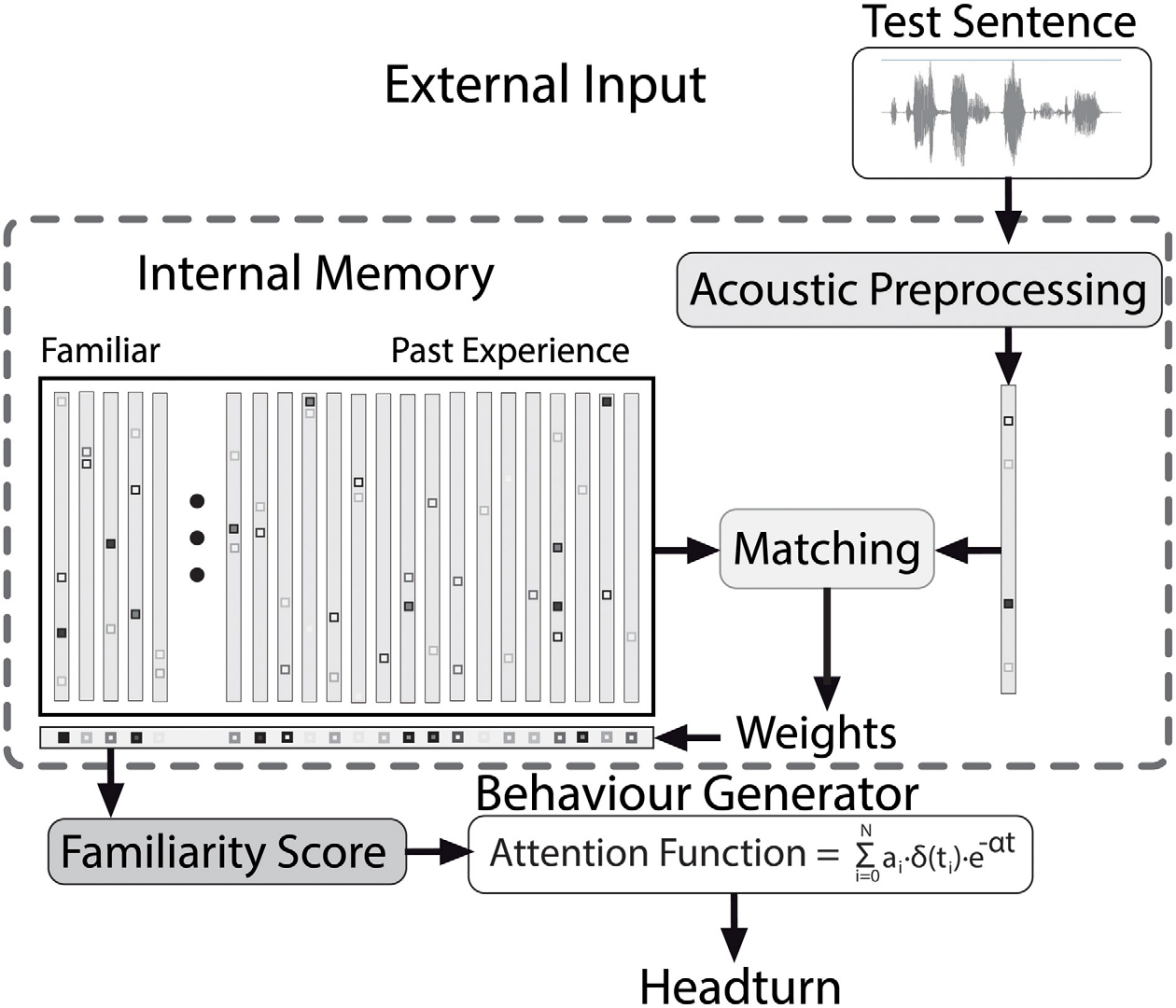
**The Headturn Preference Procedure model during the test phase, with processing stages and flow of information from external input (top) to overt behavior (bottom)**.

The focus in the present paper lies on applying the model to the test situation, as depicted in Figure 4. During the test, the model hears test sentences, which are processed and encoded in the same way as the contents of the internal memory (c.f., Section 3.2). Using the matching procedure described in Section 3.4, weights for the complete memory content are generated, which correspond to the strength of the contribution of every episode stored in the memory to processing a test stimulus. Based on the weights of the familiarization episodes and the past experience (c.f., Figure 3), a measure of recognition is computed (c.f., Section 3.5). An independent process transforms the internal familiarity score into overt behavior, as explained in Section 3.6. This allows for a direct comparison of the model output to the results of infant experiments. In the following sections we describe the model in detail.

### 3.2. Acoustic preprocessing

The processing of the acoustic speech signals starts with representing the continuous wave form in terms of its frequency and power at a given moment and the change of these properties of the speech signal over time. From the literature it appears that infant auditory processing is compatible with this form of signal processing (Saffran et al., 2007). The continuous speech signal is divided into windows with a duration of 20 ms, and for each such window a short-time spectrum is computed (Coleman, 2005). Adjacent windows overlap by 10 ms, we thus obtain 100 short-time spectra per second. The short-time spectra are converted to vectors of 13 real numbers, the Mel-Frequency Cepstral Coefficients (MFCCs), a representation that is based on knowledge about human auditory processing (Gold and Morgan, 2000). Because the auditory system is more sensitive to the rate of change in the spectrum than to static spectral features, we add the difference between adjacent MFCC vectors (known as Δ coefficients in the automatic speech processing literature) as well as the differences between adjacent Δs (known as ΔΔs). Δs and ΔΔs are vectors comprising 13 real numbers. The resulting MFCC, Δ and ΔΔ vectors corresponding to successive windows of a speech signal, are used to learn a limited number of acoustic phenomena, or prototypes. In our model we use 150 prototypes for static MFCC vectors, 150 prototypes for the Δ vectors, and 100 prototypes for the ΔΔ vectors^5^. These prototypes are used to condense the information in the MFCC, Δ and ΔΔ vectors, by representing each MFCC vector by its best matching prototype (and doing the same for all Δ and ΔΔ vectors). This converts a representation in the form of 3 * 13 = 39 real numbers to a set of three labels from a set of 150 + 150 + 100 prototypes. The conversion of the infinite number of possible MFCC, Δ and ΔΔ vectors to sets of three labels corresponds to the—admittedly unproven but plausible—assumption that audio signals are represented in the brain as sequences of acoustic prototypes.

Variable-length sequences of prototypes corresponding to an utterance must be converted to a fixed-length representation to be used in a matching procedure. For this purpose we count the number of occurrences and co-occurrences of prototypes. This results in a so called Histogram of Acoustic Co-occurrences (HAC, Van hamme, 2008). The histogram keeps a count of the number of times each of the 150 + 150 + 100 acoustic prototypes co-occurs with any prototype in its own class (including itself) at distances of 20 and 50 ms. Including co-occurrences at lags of 20 and 50 ms allows HAC vectors to capture some information about the temporal structure of an utterance. In total, a HAC vector has slightly more than 100,000 entries for all possible prototype co-occurrences. As a result, an utterance of arbitrary length, be it a single word or a complete sentence, is represented by a HAC vector of a fixed dimension. The fixed dimensionality is a requirement for most matching procedures.

### 3.3. Internal memory

Infants in HPP experiments have been exposed to speech prior to their lab visit. Therefore, the model's memory should contain some acoustic representations of past experience. Specifically, the memory contains HAC representations of a number of previously heard utterances. During the familiarization phase the acoustic HAC representations of the familiarization words are added to the memory. Therefore, the collection of HAC vectors in the memory during the test phase comprises two types of entries: the experience before the start of the HPP experiment, and the episodes the infant has stored during the familiarization phase.

The infant's experience with speech input before the lab visit is modeled by randomly selecting utterances from a corpus of infant-directed speech (Altosaar et al., 2010). Familiarization consists of adding HAC representations of tokens of two words to the memory. Although technically the model uses one single homogeneous memory, we assume that infants are able to distinguish the familiarization entries in the test from the entries from previous experience. A compelling justification for this distinction would be to assume that the familiarization utterances are stored as episodes in the hippocampus, while the previous experience is stored in the cortex (Kumaran and McClelland, 2012).

### 3.4. Matching procedure: non-negative matrix factorization

In the test phase, depicted in Figure 4, a matching procedure is necessary to compare an input stimulus to the contents of the model's memory. This matching procedure should yield scores that can be transformed into a score that corresponds to how well the representations in the memory match any particular unknown input. Episodic representations of a small number of stimuli, such as the ones the model stored during familiarization, are not straightforwardly compatible with conventional Neural Networks and similar types of Parallel Distributed Processing. Therefore, the model contains a matching procedure that is based on the assumption that the brain processes complex inputs as a non-negative weighted sum of a limited number of simpler units stored in memory. This assumption is inspired by studies on visual processing, which found that complex visual patterns are represented in primary visual cortex in the form of lines, directions, colors, and so forth (c.f., Lee and Seung, 1999; and citations therein).

Non-negative Matrix Factorization (NMF, Lee and Seung, 1999) approximates a given input (in the present simulations a HAC vector) as a weighted sum of all stored representations (here also HAC vectors) in the internal memory. Usually, NMF learns the primitives from a set of stimuli before it can be used for ‘recognizing’ unknown input, but in simulating HPP experiments we skip the NMF learning phase, and use only the decomposition mechanism. NMF can be phrased in the same terms as activation and inhibition in neural networks (Van hamme, 2011). This makes NMF, especially in the implementation that enables incremental learning (Driesen et al., 2009), a potentially interesting alternative to conventional Artificial Neural Net and Parallel Distributed Processing techniques for simulating language acquisition.

The variant of NMF used in the present paper minimizes the Kullback–Leibler divergence between a HAC-encoded test stimulus and its approximation as a positive weighted sum of all representations stored in the memory. Decoding of an unknown utterance results in a set of non-negative weights for each representation stored in the memory. The higher the weight assigned to a representation, the larger its contribution to explaining the unknown input. These weights become available immediately after the end of a test utterance^6^.

### 3.5. Recognition and familiarity scores

The matching procedure described in the previous section yields weights for all entries of the memory. The model converts these weights into a *familiarity score* that describes how well the test stimulus was recognized. The familiarity scores drive observable behavior (see the next sections). We compare two possible ways to compute familiarity scores and thereby simulate recognition.

In the first method, the familiarity score represents how much the single best-matching episode stored in memory during the familiarization phase contributes to approximating an unknown utterance in the test phase (in the presence of all other entries in the memory). This form of recognition will therefore be called *single episode activation*. In cognitive terms, single episode activation corresponds to the proposal that an infant treats the tokens of the familiarization stimuli as independent episodes that are not related to each other. This is motivated by the large acoustic differences between familiarization tokens of the same word that can be observed in the stimuli used in some HPP experiments. The second method, in which the familiarity score accumulates the weights of all familiarization entries, corresponds to the idea that the infant treats all episodes stored during familiarization as a cluster of tokens that all relate to one type of experience. This implementation of recognition will be termed *cluster activation* throughout the paper.

The scores are computed as follows: In the first implementation, the familiarity score is set equal to the *maximum* of the weights of all familiarization entries, while in the second method the familiarization score is defined by the *sum* of the weights of the familiarization entries. Both implementations of recognition yield familiarity scores that can be considered as a measure of the activation of memory representations resulting from the acoustic processing and matching procedures in the model. The familiarity score is computed independently for each test sentence. In the model we have access to the familiarity scores of each test utterance, which is evidently not possible in infants. To investigate whether familiarity scores corresponding to sentences containing a familiarized word are treated systematically differently from sentences without a familiarized word we subject the scores to independent statistical tests.

### 3.6. Behavior generation

In HPP studies, the time an infant maintains a headturn toward a flashing side lamp is measured as an overt sign of underlying attention to the speech stimuli presented via a loudspeaker on the same side. Attention is in turn driven by internal recognition. Familiarity scores, which represent cognitive processing, cannot be observed directly in infant experiments. To convert a sequence of familiarity scores to a headturn angle that varies continuously over time, our model transforms the discrete-time familiarity scores that become available at the end of each sentence in a test passage into a continuous attention function which directly drives headturns. The attention function's value at a particular time point can be interpreted as the degree to which the head is turned toward the flashing lamp and the loudspeaker. While the function value is high, the infant's head is completely turned toward the flashing lamp. As the attention value decreases, the head is more likely to be turned away from the lamp.

**Figure 5:**
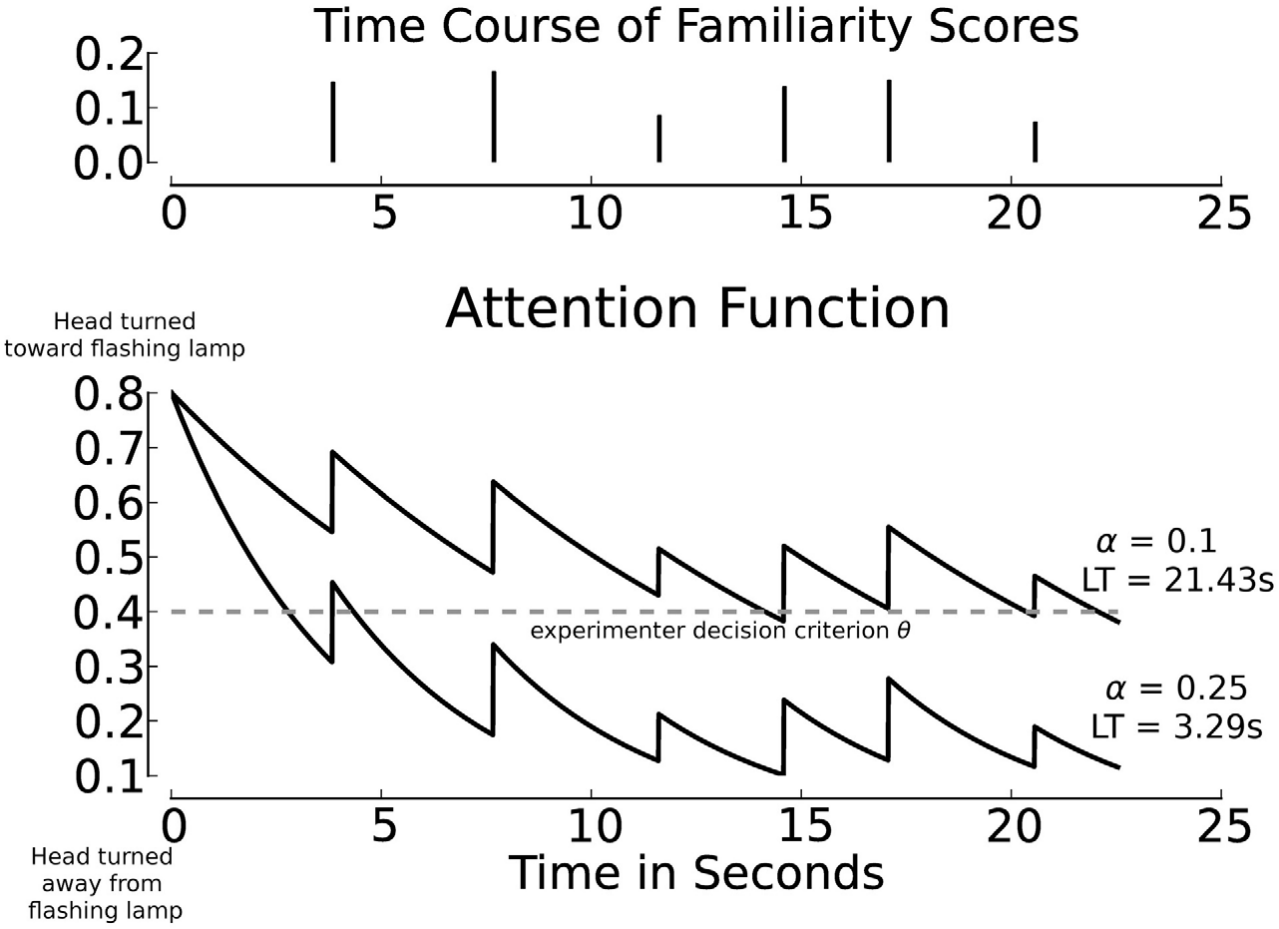
**Familiarity scores, separated by sentence duration (top panel), and exemplary corresponding attention functions (bottom panel) using grouped activations**. All material was spoken by Speaker M1. The threshold θ is set to 0.4 (dashed line), resulting listening times (LT) across exemplary values for α are annotated. In all cases the initial attention level is 0.8, which exceeds the threshold θ. The decay parameter α is independent of the familiarity scores.

In the module that converts familiarity scores into the continuous attention function, we assume that attention is renewed whenever a new familiarity score is computed (at the end of a test sentence) and that attention wanes exponentially during the course of the next sentence. The discrete-time familiarity scores are converted to discrete pulses *a*_*i*_·δ(*t*_*i*_) with an amplitude *a*_*i*_ equal to the familiarity score of the *i*th test utterance, separated by the duration of the utterances (see Figure 5, top panel, for an illustration). The sequence of pulses *a*_*i*_. δ(*t*_*i*_) is converted into a continuous function by applying an exponential decay. The resulting attention function for a passage with *N* sentences is defined as ∑^*N*^_*i* = 0_ *a*_*i*_· δ(*t*_*i*_)· *e*^−α *t*^. In this function α is a (positive) parameter specifying the decay rate, and *t* denotes time. The value of *a*_0_, the value of the attention function at the moment that the test passage starts playing depends on the value of a separate parameter ρ (see Section 3.7 for details). Figure 5 illustrates the link between pulses *a*_*i*_. δ(*t*) based on the familiarity scores (top panel) and the corresponding attention function with different values for α (bottom panel).

The decay rate α can be interpreted as the attention span of an infant. Small values of α correspond to a long attention span, while larger values of α cause the attention function to decrease more rapidly, which leads to shorter attention spans. A fixed exponential decay rate, which corresponds to an attention span that is constant for the complete duration of an experiment, is undoubtedly a strong simplification of the cognitive processes involved in converting the results of perceptual processing into observable behavior. However, there are no behavioral data that can be used to implement more complex procedures. The parameter α makes it possible to investigate whether differences in attention span between individual infants can affect the outcomes of an HPP experiment.

It should be noted that restricting a possible impact of attention span to the test phase implies that we do not model differences between infants during the familiarization phase of an HPP experiment. Effectively, the way in which we construct the memory after familiarization corresponds to the assumption that an infant pays full attention and that there are no errors in the perceptual processing. Again, this is a simplification that can only be justified by quoting a complete absence of behavioral data that would allow creating a more realistic model.

### 3.7. Simulating the test situation

In simulating the test situation, an experimenter's evaluation of infants' responses to a sequence of sentences in a test passage has to be modeled. To this end, the attention function for a passage consisting of several test sentences is assessed in a way comparable to HPP studies. In an infant study, the experimenter interprets the angle of the head relative to the center and side lamps in terms of discrete states throughout a test trial (c.f., Figure 2). The criterion that an experimenter uses to determine whether the head is turned sufficiently toward a side lamp is modeled by a threshold θ that is applied to the attention function. As long as attention exceeds θ, the head is considered to be turned sufficiently in the direction of the flashing lamp. As soon as the attention level drops below θ, the experimenter decides that the head is turned away from the lamp to such a degree that presumably the infant is no longer listening to the speech stimuli. If the value of the attention function stays below θ for more than 2 consecutive seconds, the trial is terminated (as in HPP studies).

The parameter ρ > 0 models the initial attention level above the threshold θ at the start of a test trial. It can be conceptualized as the initial degree of interest in the flashing lamp at trial onset. The value of *a*_0_, the value of the attention function at trial onset (time *t* = 0), is defined as θ + ρ, which guarantees that the infant's head is turned toward the flashing lamp sufficiently to be considered interested. In the simulations presented below, this parameter (interest at trial onset beyond threshold) was kept constant. Previous research showed that the parameter ρ does not affect the simulation results in a cognitively interesting manner (Bergmann et al., 2012). It appeared that a fixed value ρ = 0.4 was representative for the explored range of values and consequently was chosen for the present paper^7^. In Figure 5, θ and the resulting listening times obtained with two exemplary attention functions are shown. The functions are derived from the same sequence of familiarity scores (top panel); the difference between depicted attention functions and resulting listening times is due to changes in the value of α. The attention function for α = 0.25 is shown for the total duration of a test six-sentence passage. In a HPP experiment the trial would be aborted during the third sentence, because the head was turned away from the loudspeaker for more than 2 consecutive seconds.

## 4. Experiments

In the present paper, we test assumptions underlying the interpretation of HPP studies (c.f., Section 2.1), as well as two practical issues using a computational model. We briefly recall the four assumptions and explain how these are addressed in the experiments. Subsequently, we explain how the simulations address the implementation issues.

Initially, we test whether the model conforms to the assumption that test passages containing familiar and novel words yields systematic differences in internal processing and resulting listening times in two stages. In the first stage we investigate whether familiar passages yield significantly higher familiarization scores than unfamiliar passages. Thereby, we assess the model's internal ability to discriminate the two types of test stimuli. In the second stage it is tested whether the procedure that converts internal familiarization scores into overt headturns and listening times can enhance or obscure significantly different familiarization scores.

We investigate the relation between listening preference and internal recognition of the test passages by comparing two definitions of recognition (c.f., Section 3.5). In *single episode activation* the familiarity scores are based on the familiarized token in the model's memory that receives the highest weight. In *cluster activation* the familiarity scores are based on the sum of the weights of the 10 familiarization tokens in the memory. From the explanation of the model in Section 3 it will be clear that neither definition of recognition involves explicit word segmentation. If the simulations yield significant differences between test passages with familiar and with novel words, it would seem to call into question the claim that word segmentation is necessary for infants to show the observed behavior in HPP experiments. The fourth assumption that differences between individual infants do not affect the outcome of an HPP experiment will be investigated by running simulations with different values of the attention span parameter α (c.f., Section 3.6.)

In addition to the fundamental assumptions in interpreting the outcomes of HPP experiments our simulations address two implementation issues: the effects of stimulus materials and the impact of varying criteria for a sufficient degree of headturn. We run simulations with four speakers, and we will investigate familiarity scores and listening times for all combinations of these speakers in familiarization and test. By doing so, we aim to contribute to clarifying the seemingly contradicting results of previous HPP experiments on infants' generalization abilities (e.g., Houston and Jusczyk, 2000; van Heugten and Johnson, 2012). The effect of the experimenter decision criterion for a sufficient degree of headturn will be investigated by simulations with a range of values for the parameter θ (c.f., Section 3.7).

From simulations with previous versions of the computational model it became clear that many of the issues addressed above are not independent (e.g., Bergmann et al., 2012). That makes it impossible to design experiments that address one single issue in isolation. We will mitigate this problem by coming back to the individual issues in the general discussion.

### 4.1. Speech material

Our computational model requires three types of acoustic stimuli to simulate HPP studies: words spoken in isolation for familiarization, the same words embedded in continuous sentences for creating test passages, and utterances that do not contain the target words to model past language experience. All speech material in the present paper stems from a corpus of words and sentences spoken by native speakers of British English (Altosaar et al., 2010)^8^. The recordings were made in a virtually noise-free environment. Four adult speakers were available for the present study, two of whom were female.

The target words in our study were *frog* and *doll* or *duck* and *ball*. These were the words in the corpus that were most similar to the original stimuli of Jusczyk and Aslin (1995) who used monosyllabic words containing various vowels and at least one stop consonant. For each target word, five tokens spoken in isolation were available. To build the corresponding test passages, we randomly selected 24 short sentences for each of the four words. These sentences were identical for all four speakers. With these sentences a large number of distinct six-sentence test passages can be constructed by random selection.

Duration differences must be caused by different speech rates between speakers, as the sentences were identical. The mean sentence durations are between 2.69 s (standard deviation 0.33 s) for Speaker F1 and 3.0 s (standard deviation 0.39 s) for Speaker F2. The two male speakers show intermediate speech rates with 2.88 s (standard deviation 0.42 s) for Speaker M1 and 2.79 s (standard deviation 0.33 s) for Speaker M2. The range of speech rates indicates that the four speakers pronounce the same sentences at a different pace. Through the fixed time lags used to encode the acoustic input (see Section 3.2), each speaker will yield different HAC encoded vectors based on the diverging speech rates alone. We do not compensate for this source of speaker differences since there is little evidence that infants before their first birthday apply such speaker normalization (Houston and Jusczyk, 2000).

In all simulations, the internal memory consisted of 111 HAC vectors, 10 containing the two familiarized words (5 tokens for each) and 100 sentences comprising the past experience spoken by the same speaker. One additional HAC vector contained background noise (silence obtained during the recording session). The choice of 100 HAC vectors to model previous experience was motivated by exploratory simulations in which we investigated familiarity scores with memory sizes ranging from 50 to 1000 utterances to represent previous experience. Although the weights assigned to the familiarization tokens may decrease as the number of previous experience tokens increases, the relative difference between the weights of the familiarization tokens for familiar and novel test sentences is hardly affected. The NMF approximation of a test sentence will use the complete memory contents. If a familiarization token in memory is a good match for a test sentence, this is hardly changed by the number of other tokens in memory. The decision to use 100 entries for previous experience is in a sense arbitrary, but it does not crucially affect the results.

## 5. Results

The description of the results is split into two parts: First we describe the outcome of internal speech processing in the model in terms of familiarity scores. Thereby we assess the model's underlying ability to recognize familiar words in the test sentences. Subsequently, we simulate listening times and assess how the transformation of familiarity scores into overt behavior affects our results.

### 5.1. Familiarity scores

We first assess whether internal speech processing outcomes in the model can distinguish test sentences that contain familiarized words from sentences with novel words. To this end we investigate whether the familiarity scores for all 96 test sentences per speaker, used once as familiar and once as novel test item, are significantly different. For this purpose we apply the non-parametric Mann–Whitney *U*-Test. We chose this test because its efficiency is comparable to the *t*-Test with normally distributed data, while it is more robust when the data contain unequal variances or outliers.

All test sentences were recognized twice by models that were familiarized with speech from each of the four speakers. In the first recognition run the keyword in the sentence was familiar, in the second run it was novel. The whole experiment is conducted twice, once with the *single episode activation* and once with the *cluster activation* definition of recognition. Familiarity scores are computed in the manner described in Section 3.5 and are reported in percent for clarity.

#### 5.1.1. Single episode activation

Computing familiarity scores based on the single episode that receives the maximum activation yields a mixed pattern of results. The descriptive values for familiarity scores corresponding to familiar and novel test sentences can be found in Table 1. The table shows the average (and standard deviation) of the familiarity scores for all speaker pairs. Each cell contains data for the sentences in the familiar (“fam”) and novel (“nov”) condition. It can be seen that the mean values and standard deviations differ between speaker pairs. The familiarity scores are expressed in terms of the percentage of the weights of the 111 memory entries assigned to the single highest-scoring familiarization token stored in the model's memory.

**Table 1:**
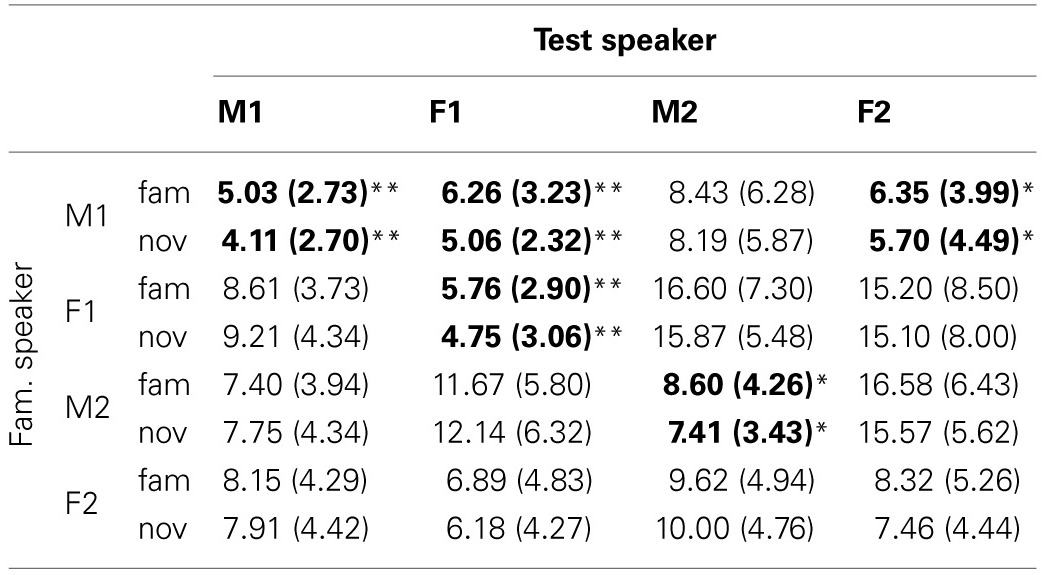
**Mean (and standard deviation) of the familiarity scores for familiar and novel sentences across speaker combinations in % with single episode activation**.

Values that differ significantly across test stimulus types are marked in bold. Significance level markers are: ^*^p < 0.05, ^**^p < 0.01.

We find statistically significant higher scores for familiar than for novel test items in five of 16 speaker pairs. Except for Speaker F2, the distinction between test conditions is statistically significant when the speaker does not change between familiarization and test. The lack of a significant difference between familiar and novel stimuli for Speaker F2 may be due to the standard deviations that are relatively large compared to the mean.

Next to the cases where the speaker did not change between familiarization and test, we see two pairs in which the test speaker was different from the familiarization speaker that yield statistically significant distinctions of familiar and novel test items. When the model has stored familiarization words spoken by Speaker M1 in memory, test sentences spoken by Speaker F1 and Speaker F2 yield significantly different familiarity scores. Interestingly, the results do not show an advantage of same-sex pairs over mixed-sex pairs.

#### 5.1.2. Cluster activation

Taking the sum of the weights for all familiarized items in memory yields statistically significant differences between familiar and novel test sentences for the four cases where familiarization and test speaker are identical, as shown in Table 2. The table is formatted in the same way as Table 1, and the values displayed refer to the percentage assigned to all 10 memory representations of the familiarized tokens. The mixed-gender speaker pairs {M1, F1} and {M1, F2} show significant differences between familiar and novel test sentences (as was the case with single episode activation). Again, we do not observe a clear advantage of same-sex pairs over mixed-sex pairs.

**Table 2:**
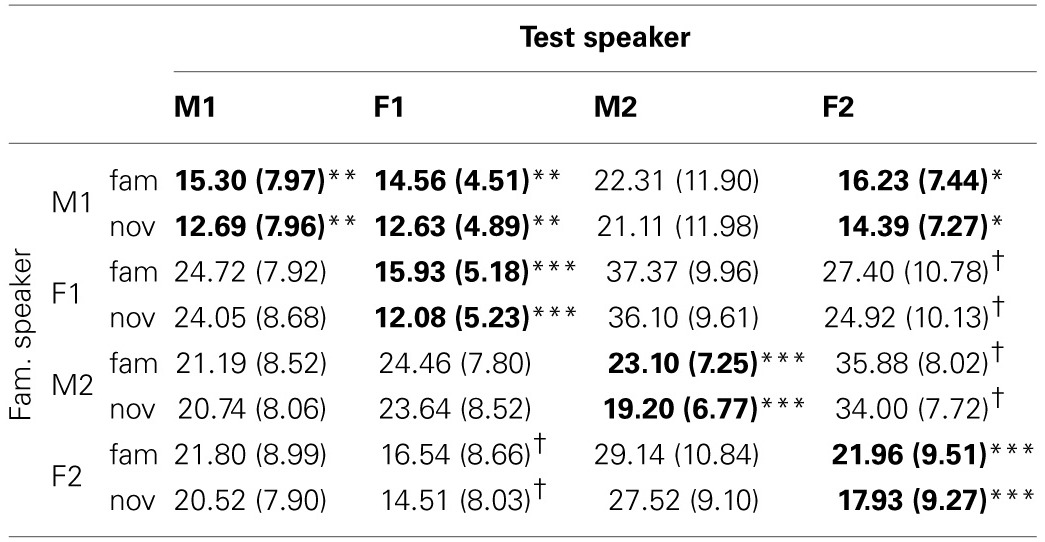
**Mean (and standard deviation) for the familiarity scores for familiar and novel sentences across speaker combinations in % with cluster activation**.

Values that differ significantly across test stimulus types are marked in bold. Significance level markers are: ^†^p < 0.1, ^*^p < 0.05, ^**^p < 0.01, ^***^p < 0.001.

### 5.2. Discussion

Overall, the model implements the assumption that processing sentences with familiar words yields higher familiarity scores than sentences with novel words, which is confirmed by the results of the simulations. The differences between familiarity scores for familiar and novel test items are larger if the speakers in familiarization and test are identical, but there is no clear effect of the sex of the speaker. The differences between the absolute values of the familiarization scores in the single episode and cluster activation runs were to be expected: sums of a set of positive numbers will always be larger than the largest individual member of a set. Perhaps the most intriguing difference between single episode and cluster activation is present when Speaker F2 utters all speech material: in the single episode activation, familiar sentences yielded no statistically significant higher familiarity scores than novel sentences, while the difference is highly significant with cluster activation.

### 5.3. Simulated listening times

In the previous section we found that our model tends to assign higher internal familiarity scores to test sentences with a familiar word than to comparable sentences with a novel word. We used these sentences to create 30 six-sentence test passages for each of the four words (*frog, doll, duck, ball*) that could be used during familiarization. Sentences were selected randomly, with replacement. Each passage contained one of the four words, which could, depending on the familiarization words, be familiar or novel. This was done for all 16 possible speaker pairs, and for the two definitions of recognition. All sequences were converted to attention functions using the procedure explained in Section 3.6, whereby we explore a range of values of the attention span parameter α. Figure 5 shows an example of one sequence, with two values of α. The value of α varied between 0.01 and 0.3, in steps of 0.01. Previous experiments with the model have shown that this range covers all cognitively relevant phenomena (Bergmann et al., 2012, 2013).

In our model, we treat the continuous attention function as identical to the headturn angle. The higher the attention function, the more the head is turned toward the side lamp (c.f., Figure 5). To compute listening times given an attention function, we need an additional parameter to model the experimenter's decision wheter the head is turned sufficiently toward the side. For that purpose we use the parameter θ explained in Section 3.7. The total listening time corresponding to a passage is the cumulated time during which the value of the attention function is above θ (counting up to the moment when the attention function is below θ for more than 2 consecutive seconds). In the simulations we varied the value of θ between 0.1 and 1.5 in steps of 0.01. Although we cannot quantify the relation between θ and the headturn angle in an infant experiment, we can say that higher values of θ correspond to stricter criteria imposed by the experimenter. Values of θ > 1.5 make the criterion so strict that most listening times become effectively zero. Very small values of θ yield listening times that are almost invariably equal to the duration of the passages.

**Figure 6:**
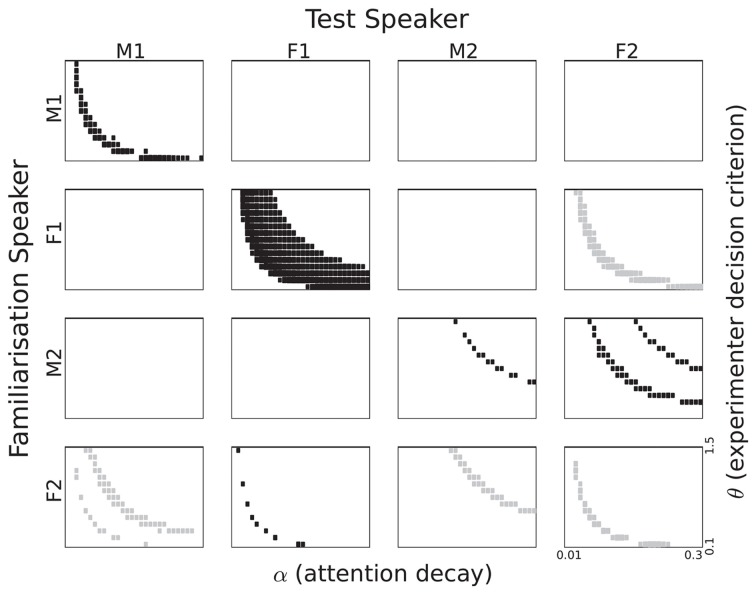
**Listening time differences for all speaker pairings based on single episode activation**. The section of the parameter space displayed corresponds to 0.1 to 1.5 for θ and 0.01 to 0.3 for α. Rectangle size corresponds to the *p*-value in a two-sample *t*-test. Black rectangles correspond to a familiarity preference, grey rectangles to a novelty preference.

**Figure 7:**
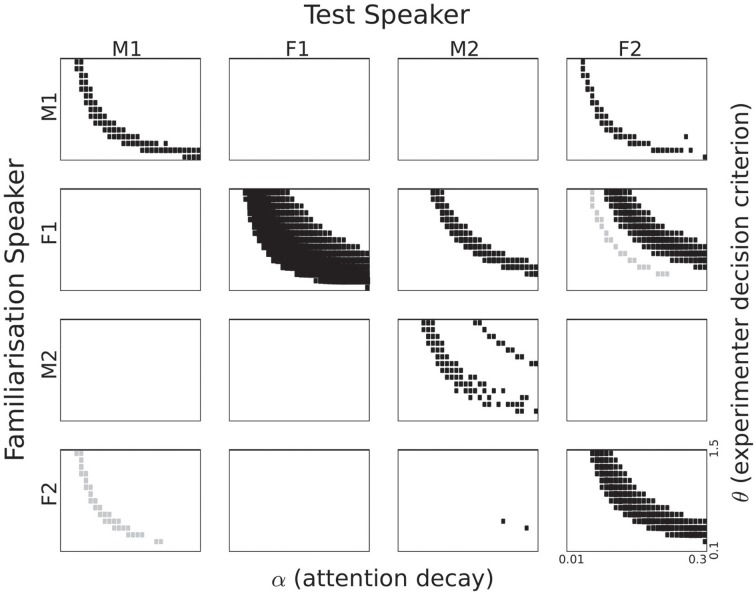
**Listening time differences for all speaker pairings based on cluster activation**. The section of the parameter space displayed corresponds to 0.1 to 1.5 for θ and 0.01 to 0.3 for α. Rectangle size corresponds to the *p*-value in a two-sample *t*-test. Black rectangles correspond to a familiarity preference; grey rectangles to a novelty preference.

To obtain an overview of the listening time differences as a function of α and θ we depict the results in the form of Hinton plots (Figures 6, 7). The figures show the {α, θ} combinations for which the listening time difference between familiar and novel passages was significant with *p*<0.05. The size of the rectangles in the figures corresponds to the significance level. If the listening time is longer for the familiar passages, the rectangles are black. Grey rectangles correspond to {α, θ} combinations in which there was a significantly longer listening time for the novel passages. *p*-values were computed using a two-sample *t*-test in which two sets of 120 passages were compared: 30 for each of the two words, which were used twice (as familiar and as novel) to remove biases caused by the fact that sentences corresponding to the words were of unequal length. We did not apply a correction for multiple comparisons for two reasons. First, it is not completely clear how many {α, θ} combinations must be included in a full comparison. For a substantial proportion of the combinations, the listening time difference is exactly zero, due to reasons that are independent of the goals of the present paper. When both α and θ are large, the attention function drops below the threshold θ more than 2 s before the end of the first sentence in a passage^9^. If both parameters have very small values, the attention function will stay above θ for the full duration of the passage. The {α, θ} pairs for which this happens might have to be excluded. One can take the position that listening time differences caused by the last sentence in a passage should also be discarded. The second reason for not adjusting the *p*-values is inspired by the shapes of the trajectories in the {α, θ} plane that can be seen in the figures. It is highly unlikely that continuous trajectories would emerge if there was no underlying process that causes the listening time differences. This procedure is similar to the procedures used in brain imaging, where the large number of comparisons between voxels would lose much of the relevant information if a straightforward adjustment would be applied, ignoring the underlying physical processes (Forman et al., 1995).

#### 5.3.1. Single episode activation

Significant listening time differences based on internal single episode activation are displayed in Figure 6 for all speaker pairings. The first thing that strikes the eye is the large difference between the four speakers. While three out of the four same-speaker pairs show a trajectory in the {α, θ} plane with a significant familiarity preference, it is also evident that the trajectory for Speaker F1 is much more robust than for the other speakers. For Speaker M2 we see a very thin trajectory. Interestingly, Speaker F2 appears to give rise to a novelty preference, despite the fact that we designed the model to yield a familiarity preference. It can also be seen that the trajectories are not always at the same area in the {α, θ} plane.

In addition to the same-speaker pairs, there are also between-speaker pairs that yield trajectories with significant differences. There is no unambiguous gender effect. The pair {M1, M2} shows no significance at all, but there are some pairings that show significant listening preferences. The patterns are not symmetric, as can be seen best for the pair M1 and F2. Familiarization with M1 gives no significant listening preferences when testing with F2, vice versa, there are substantial significant trajectories for M1 as test speaker. The lack of symmetry is perhaps most striking in the case of the two female speakers. When Speaker F1 utters the familiarization stimuli and Speaker F2 the test material, we see a novelty preference. However, when the roles are reversed between speakers a novelty preference emerges. We also see a novelty preference in the {F2, M2} pair.

***5.3.1.1. Attention span and experimenter decision criterion.*** In Figure 6 it can be seen that significant listening time differences are obtained for a wide range of values for α (on the horizontal axis), except for speaker M2. The absence of significant differences between listening times to familiar and novel passages for speaker M2 for small values of α (long attention span) is caused by the fact that the attention function never drops below the θ threshold.

Figure 6 shows an effect of the strictness with which the experimenter interprets the headturn angle, modeled by the parameter θ. For high values of θ significant listening time differences are only obtained in combination with long attention spans (lower values for α). As the value of θ decreases, significant listening time differences (both familiarity and novelty preferences) can be obtained with shorter attention spans (higher values for α). At this point we refrain from interpreting the parabolic shapes of the trajectories in the figure because a different quantization of α and θ would yield other shapes.

***5.3.1.2. Familiarity or novelty preference.*** From comparing the data in Table 1 and the patterns in Figure 6 it can be seen that there is no straightforward relation between familiarity scores for individual sentences and listening preference. Apparently, the way in which sentences are concatenated to form a passage has a substantial effect. If a sentence that yields a relatively small familiarity score is followed by a relatively long sentence, the next reset of the attention function, at the end of that sentence, may come too late to avoid the cut-off of the 2-s rule.

For some speaker pairs we see a novelty preference. Perhaps the most striking example is when the speaker F2 utters all speech material, the more so because the familiarity scores for this speaker in Table 1 suggests a familiarity preference with slightly higher values for familiar than for novel test items. However, when we base the attention function on the familiarity score of a single memory entry, it cannot be ruled out that the maximum value of a novel utterance is higher than the maximum of a familiar sentence. This can give rise to a novelty preference.

#### 5.3.2. Cluster activation

The significantly different listening times as a function of the two parameters α and θ for the cluster activation definition of recognition can be seen in Figure 7. This definition corresponds to the assumption that infants treat all familiarization stimuli as referring to a single concept and that they aim to detect references to that concept in the test passages. Numerically, summing over the activations of all 10 familiarization entries in the memory to compute a familiarity score should make that score less sensitive to seemingly random effects.

In Figure 7 we see a strong familiarity preference in all same-speaker pairs, even for speaker F2, for whom we found a novelty preference in the single episode activation case. Again, there is no unambiguous gender effect. The male speakers M1 and M2 share no pattern, while the relation between the two female speakers is quite complex. Perhaps the most striking effect is the clear familiarity preference for M2 as test speaker, if the familiarization speaker is F1. Again, we see that there is no straightforward relation between the sentence-based familiarity score data in Table 2 and the significant listening time differences in Figure 7.

***5.3.2.1. Attention span and experimenter decision criterion.*** Again, we see parabola-shaped patterns of significant differences in the {α, θ} plane. As α becomes larger, the decay of the attention function becomes more rapid, and a lower value of θ is needed to keep the attention function above threshold. As mentioned in the previous section, we refrain from interpreting those shapes since they depend on the quantization of the explored parameters.

***5.3.2.2. Familiarity or novelty preference.*** All same-speaker pairs now show a clear familiarity preference. Apparently, reducing the impact of individual memory entries leads to overall more homogeneous familiarity scores. These scores in turn lead to a familiarity preference in listening times across all four speakers.

When Speaker F1 is used to familiarize the model and Speaker F2 as the test speaker, we see a familiarity preference for some {α, θ} combinations, and a novelty preference for other combinations. This suggests that minor variations in attention span in combination with small changes in the strictness of the experimenter can cause the result of an experiment to switch from a familiarity preference to a novelty preference. While this might indeed happen in infant studies, it cannot be ruled out that the switch seen in Figure 7 is, at least in part, due to a property of the behavior generating module that is exaggerated by small changes in the decision threshold. The effect can be illustrated with the attention function for α = 0.25 in Figure 5. If the first familiarity score would have been slightly larger, the duration of the time interval where the function is below the threshold θ might have become less than 2 s. If the familiarity score for the second sentence would have been higher, listening time would increase (even if the two-second rule would have cut off the experiment during the course of the third sentence in the passage). The same effect can be caused by small changes in the threshold θ. This can be observed in the simulations with familiarization stimuli from Speaker F1 and test passages from Speaker M2.

**Figure 8:**
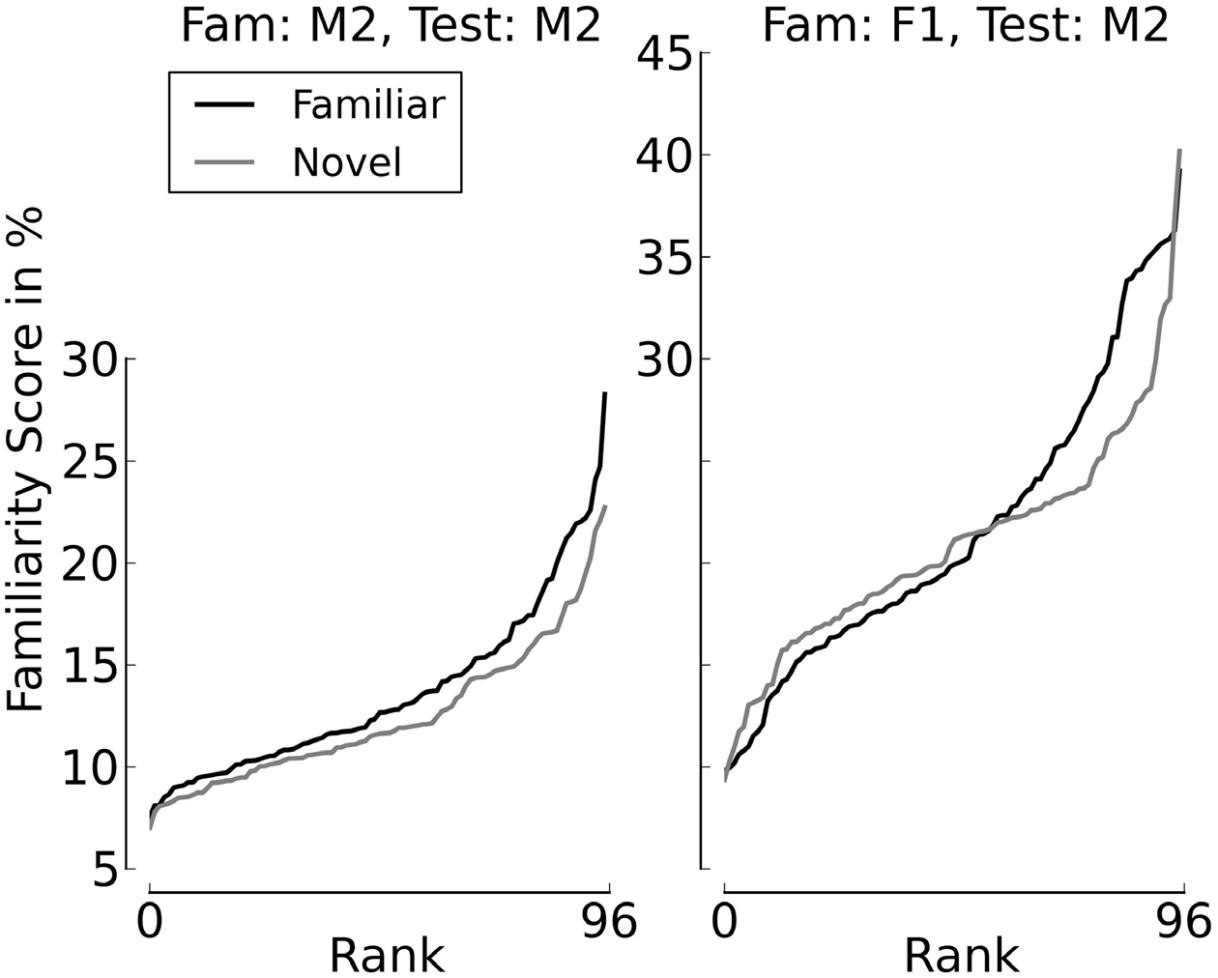
**Familiarity scores for familiar and novel test sentences, sorted by rank**. The left panel depicts a clear familiarity preference. In the right panel, the preferences cross, with lower ranks showing a novelty preference.

**Figure 9:**
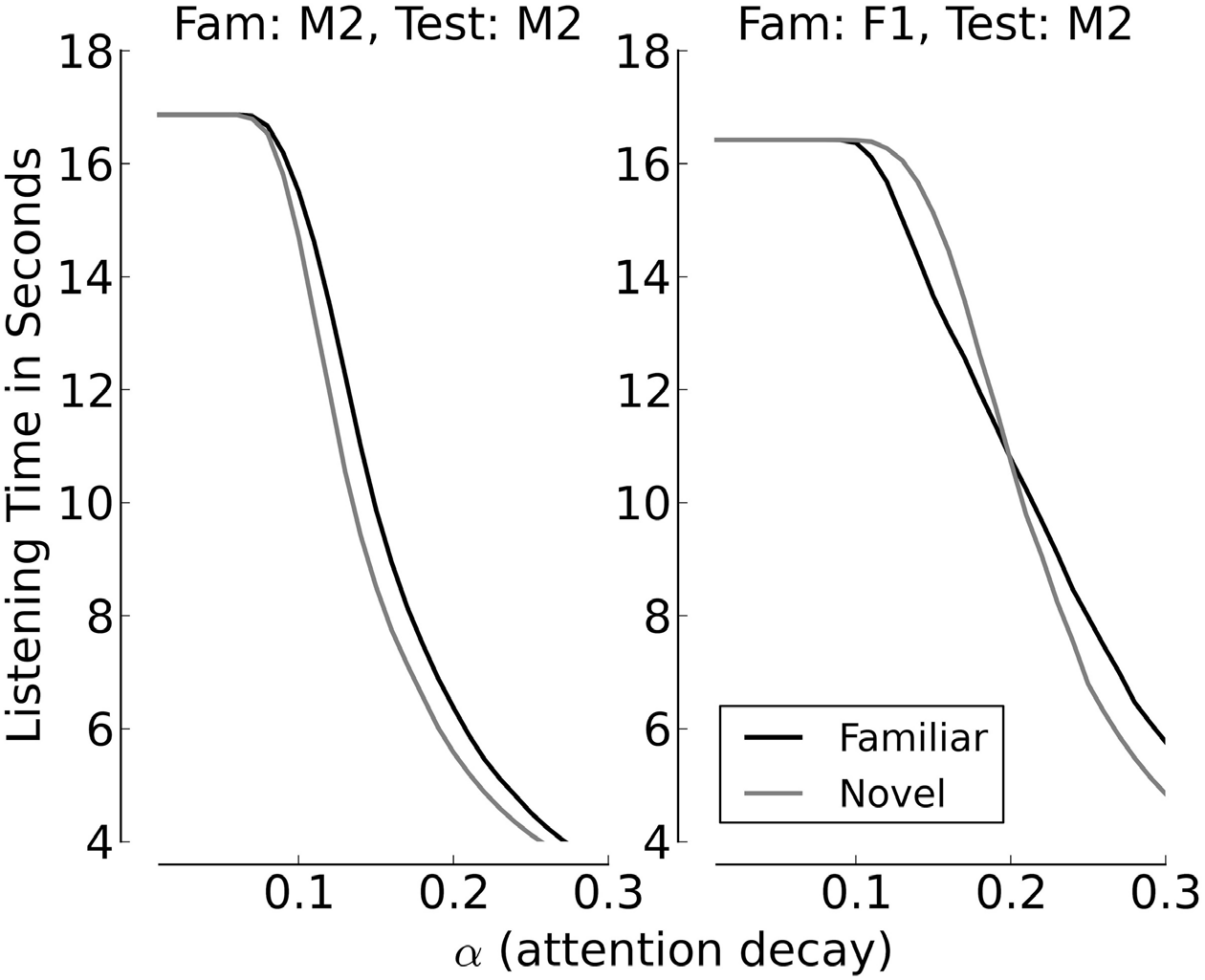
**Listening times (in seconds) across the whole range of values for α, with θ = 0.3**. The left panel shows the listening times when the same speaker, M2, utters familiarization and test stimuli, the right panel shows listening times and when Speaker F1 utters the familiarization stimuli and Speaker M2 the test items.

Figures 8, 9 provide additional support for the observation that small differences in familiarity scores, combined with specific values of α and θ, can result in switches between familiarity and novelty preference in our model. Figure 8 shows the cumulative distributions of the familiarity scores of the sentences spoken by Speaker M2 if the familiarization Speaker was M2 himself (left panel) or F2 (right panel). It can be seen that when all stimuli stem from Speaker M2, the familiarity scores are slightly but systematically higher for familiar test sentences. This is different when F1 is the familiarization speaker. As long as the familiarity scores are low, the scores for novel sentences are slightly higher than the scores for familiarized sentences. When the familiarity scores get higher, we see a cross-over point, where the familiarity scores for the familiarized utterances become larger than the corresponding scores for the sentences in the novel condition. Figure 9 depicts listening times to familiar and novel test sentences for two example speaker pairs (the same as in Figure 8) as a function of α with the assessment threshold θ set to 0.3. It can be seen in the left panel that the systematically lower scores for the novel sentences yield accordingly longer listening times in the familiar test condition for the whole range of values for α where listening time is not identical to the full duration of a passage. The right panel of the plot shows a novelty preference for longer attention spans, which switches to a familiarity preference as the value for α increases.

Figure 9 furthermore illustrates the general effect of α on the total listening time to novel and familiar passages. For small values of α, where the attention span is long and the attention function decays slowly, the total listening time is equal to the average total duration of the passages (six sentences with an average duration of slightly less than 3 s). As the value of α increases, which means that the attention span shortens, listening times decline. This is caused by a shift of the time point when the attention function drops below θ.

## 6. Discussion

In the present paper, we investigated four assumptions in the interpretation of experiments that use the HPP, a behavioral method to tap into infants' speech processing abilities. In addition, we investigated two implementation issues that may affect the outcomes of such experiments. Because the four assumptions are difficult to address in infant studies, we took recourse to computational modeling. To this end, we built a computational model that can simulate infant behavior (headturns) observed in HPP studies. The simulations address infant studies which investigated whether infants process test passages that contain words with which the infants were familiarized differently than similar passages that contain novel words (c.f., Jusczyk and Aslin, 1995).

Our model comprises several modules that operate in sequence, in a strict feed-forward architecture. We opted for this modular architecture because it enables us to investigate several processes that have been implicated in the interpretation of HPP studies in isolation. Most importantly, our model makes a distinction between the perceptual processing of the speech stimuli and the process that converts the result of perceptual processing into overt behavior. In addition, the model contains a component that simulates the decisions of the experimenter in HPP studies. Perhaps with the exception of the strict modularity and feed-forward architecture, we put a strong emphasis on making the model as cognitively plausible as possible. It processes real speech that is represented in a way we believe is neurally and cognitively defendable. The implemented matching procedure also can claim cognitive plausibility, if only because it can be combined with learning procedures that can operate in a strictly incremental and causal procedure, in which each input stimulus is used once (instead of iterating multiple times over a corpus of training stimuli).

The basic assumption in HPP studies is that different behaviors are caused by different results of processing the test stimuli. A second assumption in interpreting HPP experiments is that a listening preference for familiar (or novel) passages reflects some form of *recognition*. We defined recognition in two ways, corresponding to different hypotheses of how infants store and access familiarization stimuli during the test phase. The first definition of recognition proposes that an infant treats the familiarization stimuli as independent phenomena. In that interpretation, termed *single episode activation*, recognition was based on the single familiarization entry in the model's memory that matched a test sentence best. The alternative interpretation, *cluster activation*, corresponds to the hypothesis that the infant treats all familiarization stimuli as referring to a single phenomenon. Both definitions of recognition yielded systematic differences in the familiarity scores corresponding to familiar and novel test sentences. With cluster activation, more familiarity score differences were significant than when single episode activations were used. We believe that the larger number of statistically significant differences in the cluster activation case is, at least to a large extent, due to the fact that the sum of 10 activations is less susceptible to random variation than the maximum of a set of 10 values. Therefore, our simulations do not allow to compare the cognitive plausibility of the two interpretations of the concept of recognition.

A third assumption is that recognition of words embedded in test passages, which were heard in isolation during familiarization, implies infants' ability to segment words from continuous speech. Our model does not rely on segmentation—the division of the speech stream into smaller units, such as words. We found differences between the results of processing sentences with familiarized and novel words and we could replicate infant listening preferences using a representation of the familiarization words and test sentences that have the exact same interpretation: as a bag of acoustic events. Therefore, our model has no need for segmentation procedures. Of course, the simulations do not prove that infants do not segment the speech input, but the experiments show that segmentation skills are not necessary to solve the task posed in the type of HPP studies modeled in the present paper following the work by Jusczyk and Aslin (1995).

In the present paper we do not address studies in which passages were used for familiarization, such as the work by van Heugten and Johnson (2012). However, Jusczyk and Aslin (1995) propose that the two types of experiments are equivalent, whereas the work by Nazzi et al. (2013) indicates that there might be different processes at stake. Addressing this issue is beyond the scope of the present paper and requires further modeling work in conjunction with a careful analysis of the outcome of infant studies that use either words or passages during familiarization.

A fourth assumption in HPP studies is that differences between individual infants do not affect the outcome of an experiment, as the main comparison (listening to novel or familiar test stimuli) takes place within participants. In our model, we simulated differences between infants in the form of varying attention spans. It appeared that if internal familiarity scores distinguish the two types of test stimuli, listening time differences can emerge for a fairly wide range of attention spans. Still, the simulations show that a very short attention span can obscure different familiarity scores in the overt behavior. We deliberately kept the module that converts the results of internal processing into overt behavior very simple, and probably even overly simplistic. We did so because there are no observation data that would allow us to construct a more plausible model. Yet, our simulations show convincingly that the relation between internal processing and externally observable behavior can be complex. Behavior generation can both obscure and enhance differences in the results of internal processing and recognition. In summary, our simulations suggest that the assumption that differences between infants do not affect the results of HPP experiments should be called into question.

We explicitly modeled the experimenter's categorization of infant behavior. Our simulations show that the criterion the experimenter applies can mask listening preferences or enhance them. In addition, there is a strong interaction between the strictness of the experimenter and the attention span of the infant participants. It appeared that slightly different combinations of the factors α (attention span) and θ (experimenter strictness) can enhance or obscure listening preference and may even lead to switches between familiarity and novelty preference for some combinations of familiarization and test speakers.

We biased our model toward a familiarity preference by focusing on the parts of memory that contain the previously familiarized speech stimuli. However, in various experiments using the HPP, novelty preferences have been observed. Several suggestions regarding the cause of such a preference have been made that implicate developmental or methodological factors (Hunter and Ames, 1988). It has been suggested that individual infants differ in their general input processing strategy (Houston-Price and Nakai, 2004). Novelty preferences might arise from a focus on aspects of the input that are not captured by what has been heard most recently. In our model, different processing strategies can be implemented by changing how familiarity scores are computed from the activations of the memory contents, or from how the familiarity scores are converted to observable behavior. For example, we could discard familiarity scores that exceed an upper bound, treating the corresponding sentences as “more of the same” and therefore uninteresting. In a similar vein, we could assume that attention is aroused by new experiences, rather than by recognizing known things. In such a setting an infant would pay attention to novel stimuli, perhaps not to recognize, but rather to extend the memory by attending to and storing the representations of novel sentences. Alternatively, if we assume that an infant switches from *learning* mode during familiarization to *recognition* mode during test, we might de-emphasize the activations of the familiarization entries in the Hippocampus in favor of the background utterances in the cortex.

The exact source of the novelty preferences generated by our model warrants further investigation into the details of the implementation of the individual modules. The simulations reported in this paper uncovered interactions between the attention function derived from the familiarity scores and the experimenter's decision criterion. This interaction is strengthened by the way in which we compute the familiarity scores. In our model these scores are the result of a sentence-based recognition process. The result is only available after the sentence is complete. Technically, it is possible to change the HAC-based sentence recognition into a continuous-time process (Versteegh and ten Bosch, 2013), but doing so would require the assumption that the memory contains word-like representations.

The voices of four different speakers were used in the present experiments to explore whether non-linguistic properties of the signal can influence the presence of listening preferences. When the speakers did not change between familiarization and test, most familiarity scores were statistically different. Depending on the definition of recognition, the difference for Speaker F2 was or was not statistically significant. In our model it is possible to investigate the voice characteristics that can affect the familiarity scores in great detail. Characteristics that can have an effect depend on the representation of the speech signals in the model. For example, the MFCC representations used in our simulations do not explicitly represent voice pitch, which is reflected in a lack of clear gender-specific effects in our simulations. The co-occurrence statistics in the HAC-representation (c.f., Section 3.2) are sensitive to differences in speaking rate, since they operate with fixed time lags between acoustic events. In this context it is interesting to note that speaker F2 had a slightly lower speaking rate than the other speakers. In addition, HAC-representations can be sensitive to individual differences in pronunciation. The impact of pronunciation variation depends on the choice of words and passages, an issue that warrants further investigation. Pronunciation variation is a possible factor in infant studies as well. When different speakers are compared according to their accent, an extreme case of pronunciation variation, infants cannot detect words that recur between familiarization and test (Schmale and Seidl, 2009; Schmale et al., 2010). Both differences in speech rate and the possibility of pronunciation variants can also account for the model's mixed abilities to generalize across speakers.

Based on the investigation of the HPP in the present paper, we can make a number of predictions and recommendations for infant research. First, to faithfully measure infants' underlying speech processing abilities, it is helpful to consider their individual attention span. Attention span in the visual domain has been found to positively correlate with language development (Colombo, 2002; Colombo et al., 2008). Measuring individual attentional capabilities can thus at the same time shed light on infants' linguistic development and on an individual factor influencing their performance in HPP studies. Second, carefully defined testing procedures are necessary to allow for consistent and comparable assessments. While it is common practice within labs to have standardized procedures, there is only little exchange of precise assessment criteria across infant laboratories. For greater comparability of published results, a common assessment standard seems to be crucial. Third, an exchange of stimulus material to disentangle the properties of the speakers' voices from language-specific developmental pathways can help shed light on the factors in the stimulus material that can determine the outcome of HPP studies (Nazzi et al., 2013). Existing results using only one or a few speakers do not allow for general statements about the influence of speaker characteristics in HPP studies (c.f., Houston and Jusczyk, 2000; van Heugten and Johnson, 2012).

In summary, modeling the HPP illuminated the role of numerous factors that can determine the outcome of studies utilizing this method. The present paper exemplifies how modeling the task can help linking simulation results of presumed underlying cognitive abilities to overt infant behavior that can be measured experimentally.

### Conflict of interest statement

The authors declare that the research was conducted in the absence of any commercial or financial relationships that could be construed as a potential conflict of interest.

## Footnotes

^1^For a detailed description of the HPP, see Section 2.

^2^The HPP has also been used to investigate infants' ability to discover regularities in auditory input (see Frank and Tenenbaum, 2011; for a summary of studies in that field). However, these studies generally use artificial speech and require monitoring of a continuous monotone speech stream, arguably a different task from the segmentation studies conducted following the work of Jusczyk and Aslin (1995).

^3^Some HPP studies familiarize with paragraphs of continuous sentences and test with words in isolation, but in the present paper we focus on the predominant set-up.

^4^Jusczyk and Aslin (1995), Experiments 1–3 of 4.

^5^We used about 30 min of speech produced by two female and two male speakers of Dutch to learn the prototypes.

^6^To allow for comparisons between the decoding of different utterances, the weights obtained after each stimulus are normalized to sum to one.

^7^Increasing or decreasing the initial interest modeled in ρ shifts the overall outcome within the parameter space of {α, θ}but does not impact the general outcome.

^8^The speech material will be made available through The Language Archive at tla.mpi.nl.

^9^Up to the end of the first sentence in a passage the attention function depends only on the decay parameter α. The familiarity scores only take effect after the end of an utterance.
